# Acute Achilles Paratendinopathy following Major Injury of the Crural Fascia in a Professional Soccer Player: A Possible Correlation?

**DOI:** 10.1155/2016/1830875

**Published:** 2016-05-08

**Authors:** Gabriele Mattiussi, Michele Turloni, Pietro Tobia Baldassi, Carlos Moreno

**Affiliations:** Medical Staff, Udinese Calcio SPA, 33100 Udine, Italy

## Abstract

*Background*. The anatomy and mechanical properties of the Crural Fascia (CF), the ubiquitous connective tissue of the posterior region of the leg, have recently been investigated. The most important findings are that (i) the CF may suffer structural damage from indirect trauma, (ii) structural changes of the CF may affect the biomechanics of tissues connected to it, causing myofascial pain syndromes, and (iii) the CF is in anatomical continuity with the Achilles paratenon. Consistent with these points, the authors hypothesize that the onset of acute Achilles paratendinopathy may be related to histological and biomechanical changes of the CF.* Case Presentation*. A professional male football player suffered an isolated injury of the CF, interposed between the soleus and medial gastrocnemius (an atypical site of injury) with structural connective integrity of the muscles. After participating in the first official match, two and a half months after the trauma, he has unexpectedly demonstrated the clinical picture of acute Achilles paratendinopathy in the previously injured limb.* Conclusions*. Analysis of this case suggests that the acute Achilles paratendinopathy may be a muscle injury complication from indirect trauma of the calf muscle, if a frank and extensive involvement of the CF were to be ascertained.

## 1. Introduction

The distal myotendinous junction of the medial gastrocnemius (MG) is the typical site for muscle injuries to the calf. This particular injury is also known as a “tennis leg” calf injury and is widely known and extensively written about in literature [1].

Balius et al. [2] have recently described the injuries of the soleus (SL). The authors emphasise the fact that the rate of accidents against this muscle is likely to be underestimated, due to the anatomical complexity of the muscle and due to the low sensitivity of the ultrasound examination to detect abnormalities [3].

**Figure 1:**
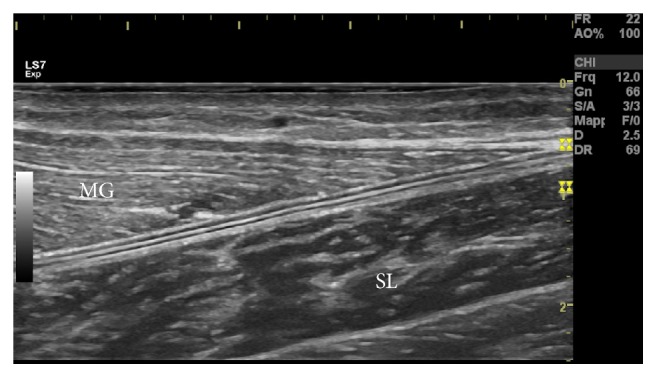
Ultrasound image of Crural Fascia (CF) in a 24-year-old male volunteer. The CF is easily distinguishable by the epimysia of the medial gastrocnemius (MG) and soleus (SL) being between these three structures (hyperechoic) interposed by two layers of hypoechoic connective tissue.

The involvement of the Crural Fascia (CF) is also possibly underestimated. It is the ubiquitous connective tissue of the posterior region of the leg that interfaces and connects the calf muscles. The CF does not integrate into the connective tissues forming the skeletal muscle extracellular matrix [4]. The CF is viewable through the use of ultrasound (Figure 1) and appears as a thick lamina of connective tissue similar to an aponeurosis [5]. The mean thickness of the (superficial) CF is estimated to be 1.1 mm in healthy subjects [6].

The close anatomical relationship of the CF with the Achilles paratenon (AP) has recently been described. The CF and AP join together at about 4 cm proximal to the posterior superior calcaneal tubercle [7, 8]; Webborn et al. [7] were also the first to describe the acute tear of the CF at the attachment to the Achilles tendon, making it an attributing factor to the etiopathogenesis of achillodynia. These new findings have a significant impact in the diagnostic study of calf muscle injury and in the evaluation of their complications.

In this study, the case of a professional football player who suffered a rare and isolated injury to the CF interposed between MG and SL is presented. The case is particularly complex because the football player, at complete sporting recovery, demonstrated the clinical picture of acute Achilles paratendinopathy in the same leg. Paratendinopathy is defined by inflammation and/or degeneration of the paratenon. Exercise-induced pain and local swelling around the tendon's mid-portion are the most important symptoms [9].

In light of the recent findings above, the hypothesis of the authors is that acute Achilles paratendinopathy may be related to the previous injury of the CF, representing a complication. To the best of the authors' knowledge, this possible correlation has never been presented in literature.

## 2. Case

### 2.1. Acute Calf Injury

A male professional football (soccer) player suffered an injury to his right calf muscle during an official match. He described his injury mechanism as a sudden “kick” from the back in his calf during a jump. He had to leave the game. On initial examination, he had localised tenderness at the middle of his right calf. Passive and active movement of the ankle exacerbated the pain. No palpable defect was noted in the gastrocnemius muscle mass. The Achilles tendon was freely movable and Thompson test was negative for Achilles tendon tear. No other injury was reported and the initial clinical diagnosis of gastrocnemius strain was established. After elastic taping in neutral ankle flexion he was sent for an emergency MRI investigation. The MRI reported very generally “gastrocnemius and soleus muscle strain with presence of fluid between the two muscles.”

**Figure 2:**
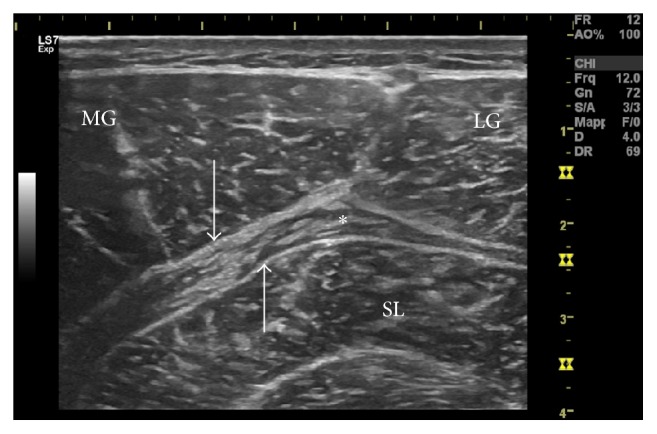
Isolated injury of the Crural Fascia, with considerable thickening of same (*asterisk*). Note the structural integrity of the medial gastrocnemius (MG) epimysium and of the soleus (SL), indicated by the* arrows*, and the absence of intramuscular edema. The muscle belly of the MG and Lateral Gastrocnemius (LG) are observable, a sign that the injury is not at muscle-tendon junction level.

**Figure 3:**
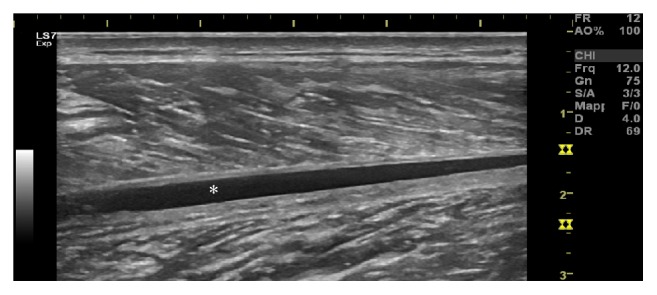
Interfascial blood (*asterisk*), observed at the distal region of the injury.

Two days later, an ultrasound examination was performed to assess what structures were actually damaged. All the ultrasound examinations presented in this study were carried out using an ultrasound GE Logiq S7 Expert (GE Healthcare, Milwaukee, WI) with a 50 mm linear footprint matrix probe (5–15 MHz). Contrary to what was expected, the pictures show a rare non-tennis leg calf injury: the epimysia of the MG and of the SL were, in fact, ecostructurally intact. However, a considerable enlargement of the connective component interposed between the muscles was observed, compatible with structural injury and retractions of the CF (Figure 2). The framework was aggravated by the presence of an extended interfascial spillage, probably blood (Figure 3). The diagnosis of the CF injury was finally made. A second MRI, performed 5 days after the trauma, confirmed “signs of detachment of the area between the SL and MG muscle in distractive outcome, with moderate intrafascial hematoma.” Another two MRIs were performed at one month and at two months after the trauma. The report of these does not add anything significant to the diagnostic study of the injury. The MRI examinations were performed using the Hitachi, Open, 2009 (0.4 T) device and interpreted by an operator with considerable experience (nonauthor contributor).

**Figure 4:**
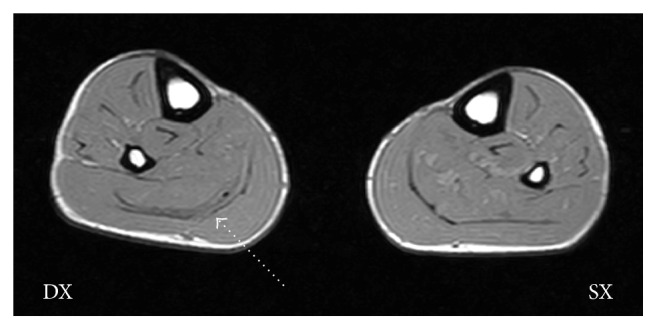
MRI axial section, performed two months after the injury. The* arrow* shows the thickening scar of the right Crural Fascia. Within the scar tissue, intrascar liquid residue, visible as a black dot, is detectable.

The recovery process is divided into three parts. The proposals of each of the stages are summarised in Table 1. The injury being very rare and its prognosis being unpredictable, we have performed over twenty ultrasound examinations during the recovery process. In such cases it is advisable to monitor the evolution of the healing process as frequently as possible; continuous monitoring has been helpful to better orientate the therapeutic proposals. The player had his first training session with the team two months after the trauma. Despite physiotherapy proposals, the healing process was completed by the formation of a hypertrophic reparative scar tissue; the presence of intrascar liquid still remained modest (Figure 4). The player complains, in particular during the last three weeks of recovery, of a feeling of excessive stiffness of the calf and Achilles tendon: sprinting and jumping were the activities of which he complains having more difficulties.

### 2.2. Acute Achilles Paratendinopathy

Fifteen days after fully returning to sports (see timeline, Table 2), the player has played in his first official match. On the day after, he appeared to be limping. At the interview, the player reported that, during the game, he felt a gradually increasing pain to the right Achilles tendon, so much so as to be forced to “run on his heel” in order to feel less pain. In any case, he played the entire match. He concludes that, on the evening of the game, the tendon felt “stiff and hot,” making it difficult to “get to sleep.”

**Figure 5:**
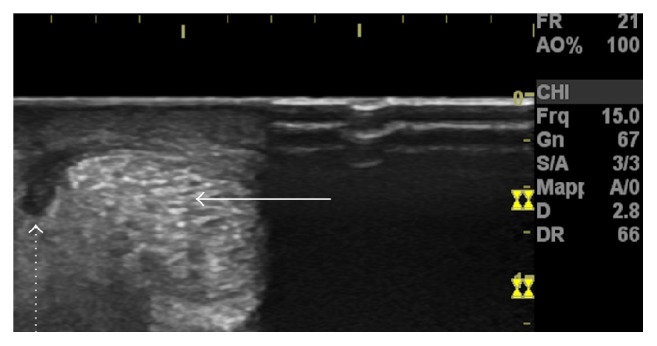
High resolution cross-sectional ultrasound of the Achilles tendon suffering from acute paratendinopathy. Laterally to the body of the Achilles tendon (*filled arrow*), a hypoechoic area is visible (*dotted arrow*), which is compatible with fluid collection between the tendon and the paratenon.

On clinical examination, the tendon appeared evidently swollen and sore to the touch, especially when pinching the side surfaces. The swelling was palpable. Consistent with the classification of the Achilles tendon related disorders presented by van Dijk et al. [9], clinical diagnosis of acute Achilles paratendinopathy was made. The picture was confirmed through ultrasound examination (Figure 5). The player suspended activity for only 3 days but therapeutic intervention (manual drainage and cryotherapy; anti-inflammatories for a week) lasted for 21 days. The player, 2 months from the onset of the pathology, did not complain of symptoms but moderate swelling of the tendon remained. The follow-up was interrupted when the player was transferred to another club.

### 2.3. Clinical History

The footballer had already suffered from acute Achilles paratendinopathy 9 months before the injury to the calf (see timeline, Table 2). The pathology had occurred two days after an injection of Betamethasone Disodium Phosphate (unknown dose), given for the treatment of right retrocalcaneal bursitis (whose symptoms lasted in turn for two weeks). Because of the condition the player had suspended activity for only five days but the symptoms resolved themselves after 6 weeks. The footballer did not take any other tendinopathy-inducing drug (quinolones, statins, and aromatase inhibitors) while he was a team member.

The player did not show clinical signs or suspicion of rheumatoid disease and blood levels were normal (routine blood tests have been performed quarterly). Instrumental screen investigations (X-Ray and MRI), performed a year and a half before the injury to the calf, did not show significant anatomical changes at the right knee, ankle, and foot.

### 2.4. Informed Consent

Written informed consent was obtained from the soccer player for publication of this case report. The subject has explicitly asked to remove personal data and not to include photos that depict him. He has consented, however, to the publication of the instrumental images.

## 3. Discussion

In this study, the case of a professional football player who suffered a rare and severe isolated CF injury, interposed between MG and SL, is presented. The case is particularly complex because the football player, at complete sporting recovery, demonstrated the clinical picture of acute Achilles paratendinopathy in the same limb.

The football player had resumed activity about two months after the trauma. The healing process was completed by the formation of a hypertrophic scar tissue interposed between MG and SL. The picture corresponds in fact to the net thickened CF, whose biomechanics were believed to be almost certainly altered. On the day after the first official match in which he participated, the clinical picture of acute Achilles paratendinopathy unexpectedly presented itself.

In light of the new findings concerning the anatomy of the CF and, in particular, the close relationship of the same with the AP, it is possible to hypothesize that acute paratendinopathy represents, at least in this specific case, a complication of the CF injury.

The following points support this hypothesis. Stecco et al. [6] observed that “posteriorly (to the Achilles tendon), the CF divided around the tendon to form the paratenon; the CF divides to envelop the tendon, thus forming the tissue usually called paratenon.” In the case presented, the structural alteration suffered proximally by the CF has likely also altered the biomechanics of the AP, creating conditions for the onset of paratendinopathy. In addition to this, it should be remembered that the presence of intrascar liquid persists. Consistent with the above, it is likely that this liquid component will be progressively “accumulated” at AP level, triggering the symptoms at the time of maximum functional load (an official match). The AP was, in fact, the only region to which the intrascar liquid had direct access; the epimysium of the muscles was intact so that the liquid could not accumulate either subcutaneously (a situation not uncommon in case of “tennis leg” injuries) or deeply, with respect to the Achilles tendon. Other authors have already described the implication of the histological and biomechanical changes of the CF in the etiopathogenesis of overuse injuries in the leg, in particular of the Medial Tibial Stress Syndrome [10] and of the accessory superficial peroneal sensory nerve entrapment [11]. Thickening of the fascia and interfascial fibrosis are in fact considered a risk factor for the development of myofascial disorders and overuse injuries.

A variable to be considered in the analysis of this case, however, is the fact that the player had already suffered from acute paratendinopathy before the injury to the calf. This manifested itself shortly after use of corticosteroids in the treatment of the deep retrocalcaneal bursitis. Consistent with the work of Turmo-Garuz et al. [12], it is possible that the medication has spread to the Achilles tendon and connected structures, in particular to the AP. If this possibility were indeed real, it would be justified to say that the injection brought on, as a complication, the paratendinopathy, potentially altering in a definitive way the histological properties. The same authors, in fact, conclude by saying that the “risk-benefit has to be taken into account when corticosteroid injections are prescribed to professional and high-level athletes” [12].

Therefore, it would be possible to hypothesize that acute paratendinopathy suffered after the calf injury is not dependent on the latter but is rather derived from previously occurring histological alteration (or even that it is an independent event). The opinion of these authors is that this view is potentially discredited by two considerations. The first is that, in the resolution of the (plausible) corticosteroid-induced paratendinopathy, the footballer has never actually presented the clinical picture and prepathological signs were not detected. The second consideration is that, being past nine months, one might have expected a clinical pattern of chronic paratendinopathy. However, none of the major clinical and instrumental characteristics of this disease [9] have been observed. For these reasons, the possibility that the paratendinopathy, suffered by the footballer after the calf injury, is connected to this (cause-effect relationship) seems more plausible than the other. Not in conflict with this, it is well-known that preexisting inflammation or degeneration is implicated as a major risk factor for tendon disorders. Therefore, despite the elimination of paratendinopathy symptoms, the footballer remained exposed to a relevant possibility of exacerbation in terms of his clinical profile.

This study has some limitations: (i) to better guarantee the anonymity of the professional footballer and his request of same, the authors have purposely omitted the basic data (age, height, BMI, and nationality) and the sports-specific data (role, dominant foot). The authors do not believe that this information is, in any case, relevant in the analysis of the case; (ii) the distinction between acute and chronic paratendinopathy can be assured only through histopathological examination. The diagnoses made in this study are only clinical or instrumental; (iii) the magnetic resonance equipment used does not offer high-quality images. This may have made the diagnosis difficult in spite of the examiner's experience: minor SL injuries may have remained undetected, in particular.

## 4. Conclusion

The analysis of the case suggests that the acute Achilles paratendinopathy may be a complication of calf muscle injury when there is decisive involvement of the CF. This possible correlation has not yet been hypothesized and verified in literature, probably because CF injuries do not generally come into consideration. The opinion of the authors is that the CF may have a role in the onset of painful syndromes and overuse injuries whose etiopathogenesis is still unclear today.

## Figures and Tables

**Table 1:** Rehabilitation course for the recovery of the calf muscle injury.

	Sports activity	Physiotherapy	Instrumental tests
Phase 1 (11 days)	Complete suspension of sports activity	Passive: manual drainage and cryopressure therapy. Double session daily Compulsory use of crutches, ankle-foot-orthosis and compression stockings	MRI: 5 days after trauma Ultrasound: daily

Phase 2 (24 days)	Bike (daily). Hydrotherapy (alternate days) Physical training in the gym (alternate days). Exercises without loading the injured calf	As in phase 1. Single session daily 2 times per week: diacutaneous fibrolysis (scar treatment)	MRI: 30 days after trauma Ultrasound: at least 3 times per week

Phase 3 (21 days)	Work on site. Progressive loads. Sports specific technical reeducation from the 45th day after injury	As in phase 2 Once per week: intratissue percutaneous electrolysis (scar treatment)	MRI: 60 days after trauma Ultrasound: at least once per week

**Table 2:** Timeline. Summary of the relevant player's clinical history.

Time interval		Pathology	Details	Evaluation
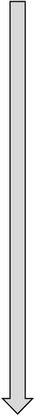	2 weeks	Retrocalcaneal bursitis	Resolution of the condition through local corticosteroid injection	Ultrasound examination
	6 weeks	Acute Achilles paratendinopathy	Clinical picture manifested itself 2 days after injection into the bursa	Clinical assessment and ultrasound examination
	9 months	The footballer trains and plays regularly		
	2 months	Injury to the Crural Fascia (rehabilitation course, described in Table 1)	Crural Fascia injury result: hypertrophic scar tissue	See Table 1
	2 weeks	The footballer trains and plays regularly		
	3 weeks	Acute Achilles paratendinopathy	Clinical picture manifested itself 1 day after the first official match in which he participated	Clinical assessment and ultrasound examination
